# Loneliness and depressive symptoms in institutionally isolated Polish adolescents explored through network psychometrics

**DOI:** 10.1038/s41598-025-06541-5

**Published:** 2025-07-01

**Authors:** Paweł Grygiel, Anna Dąbrowska

**Affiliations:** Jagiellonian University in Kraków, Gołębia 24, Kraków, 31-007 Poland

**Keywords:** Loneliness, Depressive symptoms, Network analysis, Adolescents, Institutional isolation, Psychology, Risk factors, Quality of life

## Abstract

This research investigates the link between depression and loneliness in 311 adolescents (87.8% boys; *M*_age_=15.8) in Polish Youth Correctional Centres. It focuses on identifying depression symptoms associated with loneliness, understand the centrality of loneliness in the depression symptoms network and examine the effect of various loneliness measurement methods on this relationship. The study reveals that loneliness is directly related to four affective depression symptoms: sadness, worthlessness, anhedonia and restlessness, which subsequently influence somatic symptoms. Unlike in prior research, loneliness has the least central role in the depression symptom network. The loneliness measurement method (direct/indirect) does not significantly impact its relationship with depressive symptoms. The study suggests using a simple loneliness question in network research, which is less burdensome for respondents, and discusses the reasons for the low centrality of loneliness in the network. The results underscore the significance of emotion regulation interventions for isolated adolescents’ well-being.

**Supplementary Information:**

The online version contains supplementary material available at 10.1038/s41598-025-06541-5.

## Introduction

### Depression, interpersonal relationships and loneliness

Major depression is a common^1^ and increasing^2^ mental health problem. Its affects people of all ages, also in adolescence^3,4^. Adolescent depression has multiple causes^5^ with interpersonal relationships playing a significant role in its development^6,7^.

Peers can provide both positive and negative experiences^8,9^. Positive relationships offer adolescents a sense of belonging, companionship, intimacy and emotional support^10–12^. They help counteract stress^13^boost self-esteem^14^ and self-worth^15^reducing psychological and social problems^16^. On the other hand, chronic negative peer experiences can act as stressors^17^ and lead to various disorders^18,19^including feelings of social isolation and therefore loneliness^20,21^.

Loneliness, an unpleasant and stressful emotion marked by sadness and anxiety^22–24^. It arises from a deficit between expectations and the actual or perceived state of one’s relationships^10,25–27^. It reflects an unfulfilled need for closeness^28^ and is a subjective experience, distinct from social isolation, which refers to the objective absence of relationships^29,30^.

Subjective feelings of isolation are linked to various disorders in both the elderly^31^ and adolescents^32,33^. Among young people higher levels of loneliness are associated with aggressive behavior^34^health risk behaviors such as substance use^35^physical health issues^32,33^and increased mortality^36^. Loneliness negatively impacts happiness^37^well-being^38^life satisfaction^39^ and has educational consequences affecting school attachment, learning motivation, and achievement^40^.

For more than four decades, empirical evidence has demonstrated a connection between loneliness and depression^41^. Meta-analyses reveal a correlation of around 0.5 in general population^42,43^with similar^44^ or even stronger^32^ relationships observed among children and adolescents. Unfortunately, it remains uncertain which depression symptoms have the strongest link to loneliness.

### Symptoms of depression and loneliness (network psychometrics perspective)

This ambiguity stems partly from the latent variable theory approach to psychopathological disorders .This approach posits that an unobservable common cause or latent construct (e.g. depression), explains the co-occurrence of observable symptoms (e.g. fatigue and worthlessness). Recently, an alternative framework, the network psychometrics approach, has been proposed^45^. This approach views psychological and psychiatric phenomena as a complex system of interacting, often reciprocally reinforcing symptoms^46^rather than the result of a latent variable^47,48^. For a detailed comparison of these two perspectives, see Kan et al.^49^.

The set of nodes (e.g. symptoms) and the set of edges (bidirectional pairwise relationships between symptoms) together define a network structure. Relationships (edges) can be direct or indirect, that is, through direct connections with other nodes. The strength of these links can also vary. Individual nodes can vary in the number and strength of their connections. As a result, their role in the network can also vary ‒ they can be more or less central. As certain symptoms may be of greater significance within the network, a symptom-focused approach (network psychometrics) can help determine their clinical relevance and explain the origin and persistence of mental disorders^50^. Additionally, the network approach makes it possible to identify symptoms more strongly connected to external factors (e.g., loneliness), which helps to understand the mechanisms linking broader constructs such as depression and loneliness.

Considering the role that depression and loneliness play in the development of adolescents, it is interesting how little attention has been paid to their network relationships. To our knowledge, only ten studies (in nine articles) address this question^51–59^.

The results generally highlight loneliness as a key factor in the network of depressive symptoms. In six out of ten analyses, loneliness ranks as the most or second-most central network element^53^ or second largest central network element^51–59^. Its centrality never falls below the median for all elements of the network, suggesting a strong association with depressive symptoms in adolescents and a potential risk factor for the development of the disorder.

These studies also reveal that loneliness is mainly linked to sadness, lack of happiness, crying, and suicidal ideation. It connects loneliness to depression through a negative cognitive and emotional style that activates sadness or depressed^60^ – one of the central symptoms of depression^61^. These analyses significantly improve our understanding of the relationship between loneliness and depressive symptoms.

### Limitations of previous research

Unfortunately, previous research on the relationship between depressive symptoms and loneliness is not without limitations. Above all, with the exception of the study by Kenny et al.^52^most questionnaires used to estimate the networks include, in addition to loneliness, several items related to interpersonal relationships. Some of these items are only loosely connected to loneliness. For example, the Children’s Depression Inventory (CDI) includes five such items (e.g., “Nobody really loves me,” “I do not have any friends”). Similarly, the CDI-2 also contains five items related to interpersonal relationships. The Mood and Feelings Questionnaire (MFQ) has four, and the Short Mood and Feelings Questionnaire (SMFQ) contains one (“I thought nobody really loved me”).

Centrality in a network indicates the importance of a node relative to others. Therefore, the presence of many items related to interpersonal relationships — all likely to correlate with loneliness — might artificially inflate the centrality of loneliness^50,62^. Therefore, conclusions about the high relevance of loneliness to depressive symptoms can be questioned^59^.

The inclusion of many social-related items results from a general feature of the research scales: they often go beyond depressive symptoms as defined by DSM-IV/V^63,64^ or ICD-10/11^65,66^. For example, as demonstrated by Manfro et al.^56^of the 33 items that comprise the MFQ, up to 12 (one-third) are not explicitly captured by the DSM/CDI criteria for MDD. This heterogeneity characterized not only MFQ, but also CDI and CESD^67^. The question then arises whether the networks analyzed are really networks of depressive symptoms or constructs beyond depression? Note that we do not question the utility of instruments covering a wide range of test elements, including those outside DSM or ICD^67,68^. We only conclude that capturing the relationship between loneliness and depressive symptoms will be more conclusive when analyses are based on scales without items beyond the commonly accepted criteria.

Previous research has limitations as all studies used a single self-report item directly asking about feelings of loneliness. While common in epidemiological studies^69^this approach may introduce bias due to social stigma^70–72^leading to under-reporting of loneliness^73^. Additionally, the ability to identify as lonely, especially for young people with limited cognitive abilities, can pose another challenge when using direct measures of loneliness^74^.

Another approach is using multi-item scales that do not mention “loneliness” directly (e.g., UCLA or DJGLS^41,75^. Studies show that the choice between direct and indirect measures can affect research conclusions – about 50% of respondents report loneliness directly but not indirectly^69,74^. Although direct and indirect measures produce different patterns, all previous investigations exclusively used direct measurements. In our study, we compared direct and indirect loneliness measures to determine how their relationships with depressive symptoms differ.

It should also be noted that the research to date has focused on the general population of children and adolescents. Only two studies included specific groups of teenagers: clients of the child and adolescent psychiatry clinic^57^ and left-behind children^55^. To our knowledge, no network analyzes have examined the interconnections of depression symptoms and loneliness among adolescents in institutional isolation. Recognizing the relationship between loneliness and depressive symptoms in this group of young people may be of interest for several reasons.

Several studies have shown that depression is more common in the juvenile justice system than in the general population of adolescents. A meta-analysis based on 33 studies in 18,861 adolescents estimated that major depression spreads to 10.1% among male adolescents and 25.8% among female adolescents in juvenile detention and correctional facilities^76,77^. High pooled prevalence rates (12%) were also found in adolescents placed in out-of-home care^78^. In the general adolescent population, the prevalence rate is several times lower and is estimated at 2.6% for any depressive disorder and 1.3% for major depressive disorder (MDD)^4,79,80^.

Furthermore, studies show that institutional isolation is strongly associated with experiences of loneliness. This pattern has been observed not only among adult prisoners^81–88^but also among adolescents in juvenile correctional institutions^89,90^ or residential care^91,92^. According to information provided by the Survey of Youth in Residential Placement (SYRP), collected from 7073 youth in custody in 2003, 52% of youth reported feeling lonely ‘too much of the time’^93^.

To address these gaps and move beyond global associations, the present study examines whether the symptom-level dynamics between loneliness and depression differ in institutionally isolated adolescents. Although loneliness and depression are consistently linked across adolescent populations^32,44^it remains unclear whether these associations generalize to symptom-level dynamics among institutionally isolated youth. Institutional isolation, such as placement in residential care or correctional facilities, creates a distinct social environment characterized by chronic stressors like enforced separation from prior networks, restricted autonomy, rigid external control, and stigmatization^94–97^. While, as previously noted, elevated rates of both loneliness and depression have been observed in these settings^98–100^the functional connections between specific symptoms ‒ such as whether loneliness drives sadness or somatic complaints ‒ remain unexplored.

Institutional environments may fundamentally alter loneliness–depression pathways. Prolonged isolation and lack of autonomy may impair emotion regulation, amplifying links between loneliness and affective symptoms like worthlessness or anhedonia^22,101^. Chronic exposure to rigid hierarchies may dysregulate biological stress systems (e.g., HPA axis^102^), potentially shifting prominence toward somatic symptoms like fatigue and insomnia. Moreover, limited opportunities to rebuild social networks may decouple loneliness from interpersonal mediators typically observed in community samples^87,88^.

These contextual factors suggest that the network structure ‒ not merely the correlation ‒ between loneliness and depression may differ in institutionally isolated adolescents. For instance, loneliness, typically a central node in community-based networks^51,57^could become marginalized amid competing stressors like hypervigilance or institutional routine compliance. Previous network studies may have inflated loneliness’s centrality by using depression scales with items beyond DSM/ICD criteria (e.g., interpersonal conflict^67^). By employing a DSM-aligned depression scale and comparing direct and indirect loneliness measures, this study addresses these methodological limitations and tests whether institutional isolation reshapes the symptom network’s architecture.

If loneliness shows low centrality despite strong correlations with affective symptoms, interventions targeting emotion regulation rather than social connectivity may be more effective. Conversely, if the network structure mirrors that of community samples, this would suggest broader transdiagnostic mechanisms linking loneliness and depression. Either outcome advances theory by clarifying how environmental constraints shape psychopathological processes.

### Current study

Existing research has shown a significant relationship between depression and loneliness in adolescents. However, many of these studies are based on latent variable theory, neglecting the depression network structure. Few investigations have explored the symptom-level connections between depression and loneliness. Those that have often relied on depression indicators extending beyond DSM or ICD definitions. Moreover, previous studies exclusively used direct measures of loneliness. This approach could introduce bias due to stigma associated with mental illness and limitations in adolescents’ cognitive abilities. Notably, none of these studies focused on institutionally isolated youth who are at a higher risk of depression and loneliness.

This study aimed to investigate potential functional relationships between individual depression symptoms and loneliness in institutionally isolated adolescents, using a network psychometrics analysis. Unlike previous research, we used a questionnaire (Revised Children’s Anxiety and Depression Scale - RCADS-MD) focusing solely on the defining symptoms of depression as per official classifications (ICD/DSM). We also employed both direct and indirect loneliness measures. The study had two main objectives: (1) to identify specific links between loneliness and individual depression symptoms in institutionally isolated adolescents, and (2) to assess the structural role of loneliness within the depressive symptoms network.

## Method

### Participants and procedure

The participants were 311 Caucasian adolescents (87.8% boys) from six Youth Correctional Centres (YCC; in Polish: Młodzieżowe Ośrodki Wychowawcze ‒ MOW) located in southern Poland. YCCs currently play a dominant role in in the Polish system of re-socialization for socially maladjusted adolescents^103^. They are intended only for young people (between 13 and 18 years old) for whom Family and Juvenile Departments of District Courts have applied an educational ruling in the form of placement in such centers^104^. In 2020 there were 4357 juveniles placed in 94 YCCs^105^.

YCCs are rehabilitation institutions (providing a broad scope of services directed at education, resocialization and support for juveniles) of an isolated nature^106^. These centers operate 24 h a day and young people can temporarily leave YCC for holidays, winter and summer vacation only after a positive decision of the family court. The decision on the pass is made each time on the basis of an application submitted by caretakers from YCC including, among others, a description of the progress of resocialization of the pupil^107^.

The study involved adolescents aged 13–18 (*M* = 15.8; *SD* = 1.77) with an average stay of 26.48 months (*SD* = 21.40) in YCCs. Prior to placement, 37% lived with both parents, 31% with mother only, 5.5% with father only, and 25.7% with non-biological parents. Among parents, 40% had full authority, 50% limited rights and 10% no rights. The primary reasons for placement in a YCC were demoralization (60.8%) and criminal acts (39.2%).

Data were collected at the end of 2019 and the beginning of 2020 using the computer-assisted web interview (CAWI) technique in the form of an online questionnaire on a computer/laptop by each student (in the presence of a trained interviewer/instructor). Due to the use of CAWI as a data collection technique, the database did not include the missing data. The participants did not receive any payment.

Participation in the study was entirely voluntary. Prior to the start of the study, all participants were informed about its purpose, procedures, and their right to withdraw at any time without providing a reason. Each participant gave informed consent to take part in the study. In the case of minors, consent was also obtained from a parent and/or legal guardian. The study was approved by the Research Ethics Committee of the Faculty of Philosophy, Jagiellonian University (decision no. 221.0032.6.2021). All methods were carried out in accordance with the Declaration of Helsinki and the ethical standards of the institutional research committee.

### Measures

#### Major depression—the revised child anxiety and depression scale (RCADS‑MD)

The RCADS^108,109^ is one of the most widely used self–report questionnaires for a brief screening assessment of symptoms of anxiety and depression among children and adolescents. This measure – based on the DSM-IV criteria^110^ – is composed of six subscales including measuring major depression (10 items; for example: “I feel sad or empty”, “I am tired a lot”) scored on a 4-point scale (0 = never, 1 = sometimes, 2 = often, 3 = always). The respondent is asked to report current symptoms. Higher scores indicate higher intensities of depressive symptoms. In our data Cronbach’s α was 0.90.

#### Indirect measure of loneliness—The de Jong Gierveld loneliness scale (DJGLS)

The DJGLS^75,111^ was designed as an unidimensional instrument to study a generalized feeling of loneliness. The questionnaire consists of 11 items (examples: “I often feel rejected”, “There are enough people I feel close to”) and does not contain the word “loneliness”. Interviewees respond using a 4–point scale ranging from 1 (yes! ) to 4 (no! ). After recording the negatively worded items, a higher total score indicates a higher level of loneliness. The scale is reliable and valid for adolescents^112^. In our data, the Cronbach’s α for this measure was 0.87.

#### Direct (self-rating) measure of loneliness

The direct measurement involved one item concerning loneliness: “How much do you feel lonely?” Respondents select a response on a 101-point scale (slider question) where 0 means “I don’t feel lonely at all” while 100 means “I feel very lonely”.

### Statistical analyses

#### Network estimation

We estimated networks using regularized Gaussian graphical models (GGM)^113^. This method applied a least absolute shrinkage and selection operator (LASSO)^114^combined with an Extended Bayesian Information Criterion (EBIC)^115^ model selection. This approach estimates partial correlations, excludes spurious associations, and shrinks trivial relationships to zero. As a result, the estimated network is sparser and more interpretable^116^^1^. To visualize GGM networks, we used Fruchterman and Reingold algorithm^117^. In this layout, nodes with stronger or more connections are positioned closer together, while nodes with weaker correlations are placed further from the center.

In addition, we also estimated the shortest paths between loneliness and all other variables in the network using the Dijkstra algorithm^118^. The shortest paths represent the most efficient routes connecting one node to another, either directly or indirectly. They are calculated based on the sum of edge weights along each potential route, with the shortest paths consisting of the smallest cumulative weights^119^. It is important that “even though two nodes may share a direct path, an indirect route via an intermediary node may consist of stronger associations and therefore be a quicker route”^120^. In this way, the shortest-path network may be helpful in identifying potential mediation paths.

We also assessed the importance of individual symptoms by calculating their strength centrality. Strength centrality reflects the sum of weighted direct connections between a node and all other nodes. High node strength indicates a strong potential direct influence on other nodes in the network.

Additionally, we conducted bootstrapped centrality difference tests using 1,000 resamples and an alpha level of 0.05. These tests evaluated whether certain nodes were significantly more central than others. A similar procedure was applied to test for significant differences between specific edge-weight pairs.

We used a case-dropping bootstrap procedure, resampling the network 1,000 times, to evaluate node strength stability. The procedure was summarized using correlation stability coefficients (CS-coefficients), which indicate the maximum proportion of cases that can be dropped while retaining a correlation of 0.70 or higher with the original centrality estimates (at 95% confidence). Ideally, the CS-coefficient should exceed 0.50, and minimally 0.25^121^.

Next, we compared the networks estimated using direct versus indirect loneliness measures across several dimensions. First, we tested for global strength invariance ‒ whether the total connectivity (i.e., absolute sum of all edge weights) was identical across networks. Second, we examined structural invariance by testing the maximum absolute difference across all individual edges. Third, we assessed edge strength invariance, evaluating whether specific edges were significantly different between networks. To test the differences between networks, we used the two-tailed resampling-based permutation test in which the pairwise difference between groups was calculated repeatedly (1000 times in our analysis) for randomly regrouped individuals^122^.

## Results

### Preliminary analysis

**Fig. 1:**
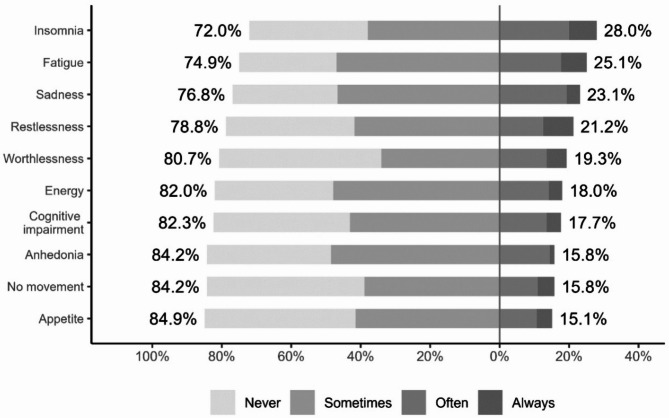
Frequency of depressive symptoms measured by the RCADS-MDD.

An analysis of the students’ responses to the question on the prevalence of depression symptoms shows a fairly significant variation (see Fig. 1). The most endorsed symptoms (defined as feeling at least „often”) of the major depression symptoms are insomnia (“I’m having trouble sleeping” – 28%) and fatigue (“I’m tired a lot” – 25.1%) (see Fig. 1). The last endorsed symptoms are appetite (“I have problems with my appetite” – 15.1%), no movement (“I feel like I don’t want to move” – 18.8%) and anhedonia (“Nothing is much fun anymore” – 15.8%).

In addition, the overall level of depressive symptoms measured with the RCADS-Major Depression subscale was moderate, with a mean score of 18.78 (*SD* = 6.10) on a scale ranging from 10 to 40. For comparison, in our unpublished dataset collected among younger adolescents (*M*_age_ = 13.56 years, *SD* = 0.76; *n* = 490) attending primary schools, the mean RCADS-MD score was significantly lower (*M* = 14.90, *SD* = 4.37). An independent-samples t-test confirmed that this difference was statistically significant, *t*(799) = 10.46, *p* < 0.001, with a medium-to-large effect size, *d* = 0.76. These findings provide preliminary evidence that depressive symptoms are substantially elevated among adolescents placed in Youth Correctional Centres compared to their younger peers attending mainstream schools.

When asked directly about feeling lonely (“How much do you feel lonely?”), participants reported a mean score of 43.36 (*SD* = 35.05) on a 0–100 scale, suggesting a moderate level of perceived loneliness. The indirect measure of loneliness (De Jong Gierveld Loneliness Scale) yielded a mean score of 24.60 (*SD* = 7.73) on a scale ranging from 11 to 44, where higher scores indicate greater loneliness. Compared to our unpublished dataset collected among younger adolescents (*M*_age_ = 13.56 years, *SD* = 0.76; *n* = 490) attending primary schools, where the mean DJGLS score was 15.64 (*SD* = 5.60), the level of loneliness among adolescents in Youth Correctional Centres was substantially higher. This difference was statistically significant, *t*(799) = 18.98, *p* < 0.001, with a large effect size (*d* = 1.38), highlighting markedly elevated loneliness levels in the correctional group.

**Fig. 2:**
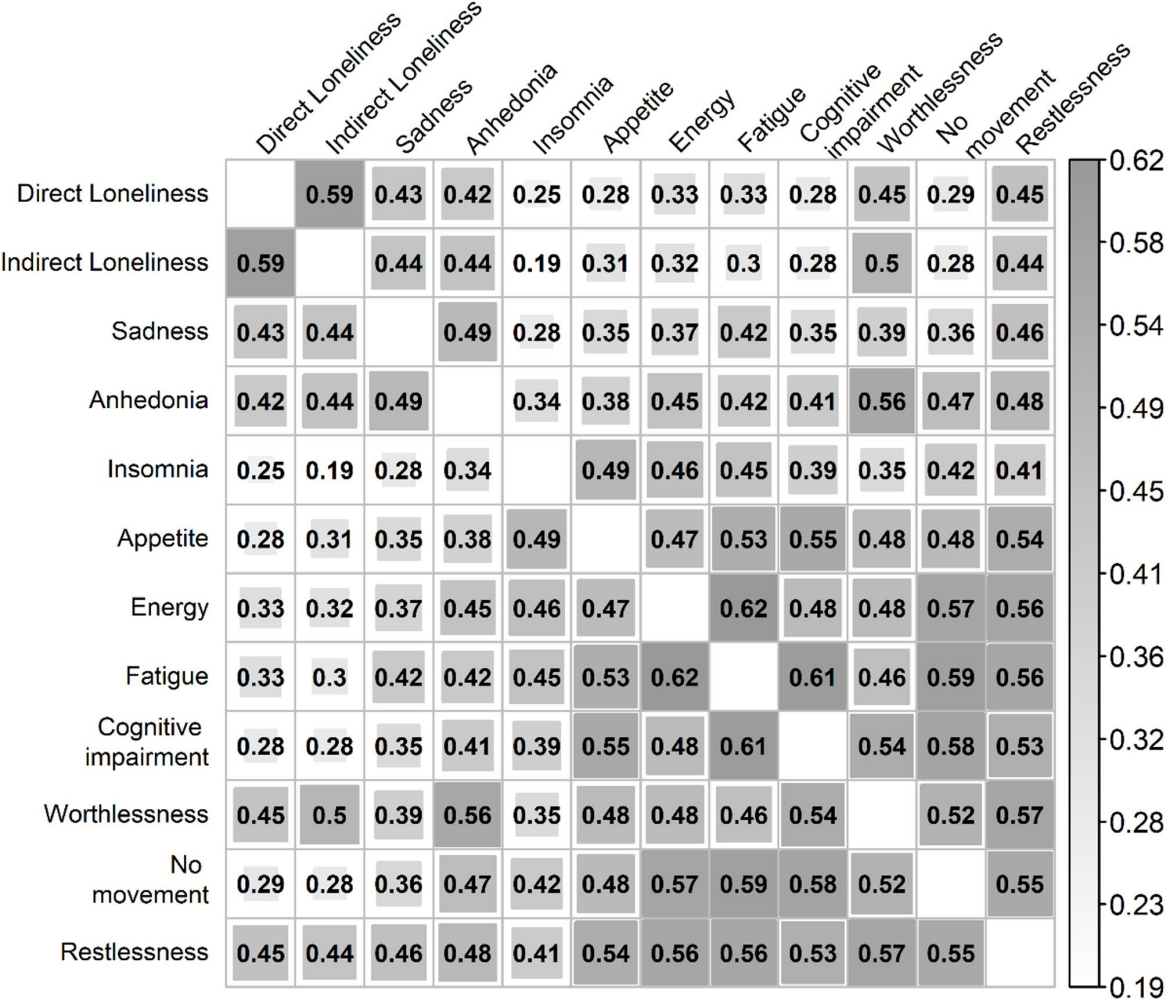
Matrix of rho Spearman correlations between the studied variables. All coefficients are statistically significant at *p* < 0.01.

The correlations between depression symptoms and loneliness (direct and indirect) are provided in Fig. 2. All correlations proved to be statistically significant at the 0.01 level of confidence.

### Main analysis

**Fig. 3:**
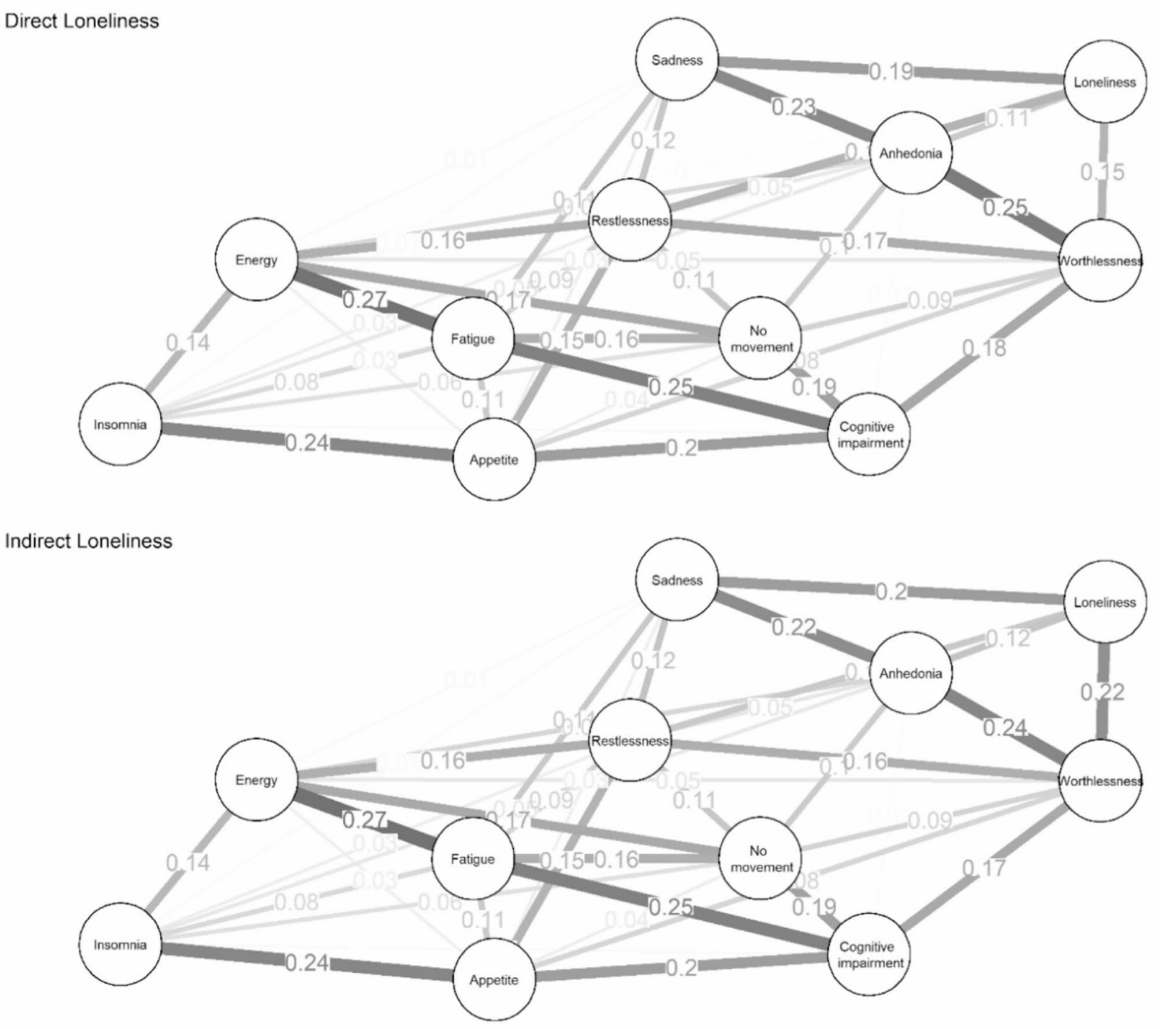
EBIC gLasso network of the RCADS-MDD symptoms and loneliness.

Figure 3 shows EBIC gLasso networks of RCADS-MDD symptoms with direct (panel A) and indirect (panel B) loneliness. Full edge weight matrices corresponding to the networks are provided in Table S1 in the Supplementary Materials. Direct loneliness had 42 non-zero edges out of 55 possible (mean edge weight: 0.087), while indirect loneliness had 41 (mean edge weight: 0.087).

To further explore these network structures, we next identified the specific depressive symptoms most closely associated with loneliness. In the network involving indirect loneliness (measured via the DJGLS scale), indirect loneliness was associated with four depressive symptoms: sadness (*rho*_*p*_ estimate = 0.20), worthlessness (*rho*_*p*_ estimate = 0.22), anhedonia (*rho*_*p*_ estimate = 0.12) and restlessness (*rho*_*p*_ estimate = 0.11). In contrast, in the network involving direct loneliness (measured via the direct self-rating question), direct loneliness was associated with five depressive symptoms: sadness (*rho*_*p*_ estimate = 0.19), worthlessness (*rho*_*p*_ estimate = 0.15), anhedonia (*rho*_*p*_ estimate = 0.11), restlessness (*rho*_*p*_ estimate = 0.15) and (additionally) energy (*rho*_*p*_ estimate < 0.01). Thus, while the core associations with sadness, worthlessness, anhedonia, and restlessness were consistent across both measures, direct loneliness showed an additional link with decreased energy levels.

To assess the robustness of these associations, we conducted bootstrapped difference tests for edge weights (see: Figure S1). In these tests, bootstrapped non-parametric 95% confidence intervals are used to assess whether the strength of one connection in the network is significantly different from another. In simple terms, they test whether certain symptom connections are truly stronger than others, or whether observed differences could result from random sampling variability.

In both the direct and indirect loneliness networks, no significant differences were found between the edge weights linking loneliness to depressive symptoms, indicating a relatively stable pattern of associations. An exception emerged for the direct loneliness network: the edge between loneliness and energy was significantly weaker compared to the edges between loneliness and worthlessness (95% CI = [-0.24; -0.01]), sadness (95% CI = [-0.29; -0.03]), and restlessness (95% CI = [-0.24; -0.01]). Notably, the loneliness–energy connection did not significantly differ from the loneliness–anhedonia connection. These results suggest that, with minor exceptions, the strength of the associations between loneliness and depressive symptoms remained consistent^2^.

To evaluate whether the strength of the correlation coefficients varies significantly between the two networks, we compared them using the Network Comparison Test (NCT). Post hoc permutation tests did not show significant differences for any of the edges through which loneliness is linked to depressive symptoms. The lowest level of significance – but exceeding the cutoff of 0.05 – was in the case of loneliness↔worthlessness (Δ_rp_ = 0.07, *p* = 0.378) and loneliness↔restlessness (Δ_*rp*_ = 0.04, *p* = 0.552) (see also Figure S2)^3^.

**Fig. 4:**
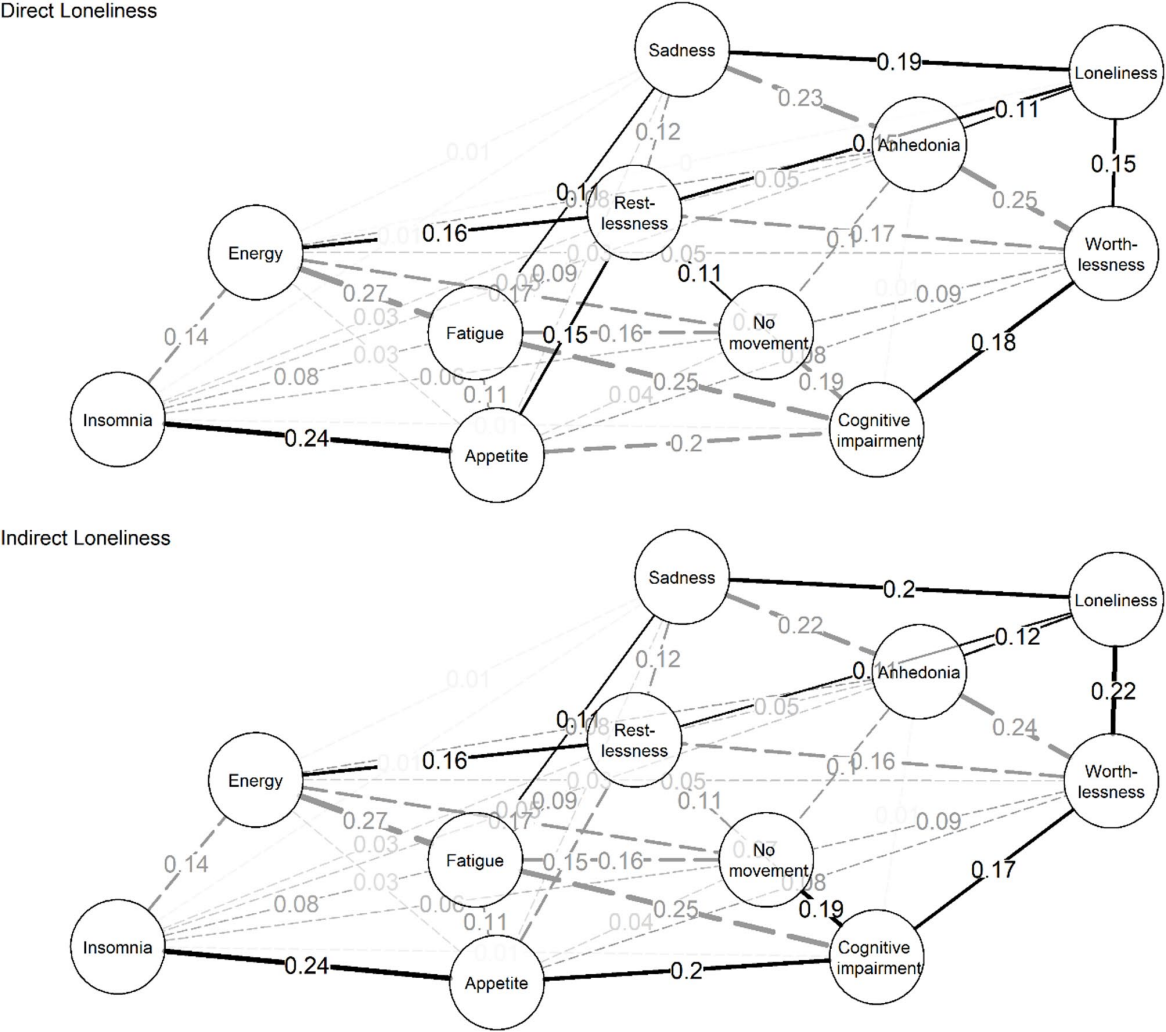
Network highlighting shortest-paths between loneliness and depression symptoms. Solid lines represent shortest paths, dashed lines represent connections that do not lie on the shortest paths. The wider the line, the stronger the correlation.

Building on these results, we next investigated how loneliness is embedded within the broader structure of depressive symptom networks by examining the shortest paths between symptoms (see: Fig. 4). In network analysis, the shortest path represents the sequence of connections requiring the least “steps” to link two nodes, indicating the most efficient route of potential influence between variables. In both the direct and indirect loneliness networks, the shortest paths from loneliness to all somatic symptoms (e.g., fatigue, energy, appetite, insomnia) consistently traverse through four affective symptoms: sadness, worthlessness, anhedonia, and restlessness. This suggests that affective symptoms act as crucial intermediaries linking loneliness to somatic experiences of depression. For example, in the direct loneliness network, even though a direct connection exists between loneliness and energy, the shortest path passes through restlessness, indicating the mediating role of affective dysregulation. These patterns underscore the central importance of affective symptoms in understanding how loneliness may relate to broader depressive symptomatology.

**Fig. 5:**
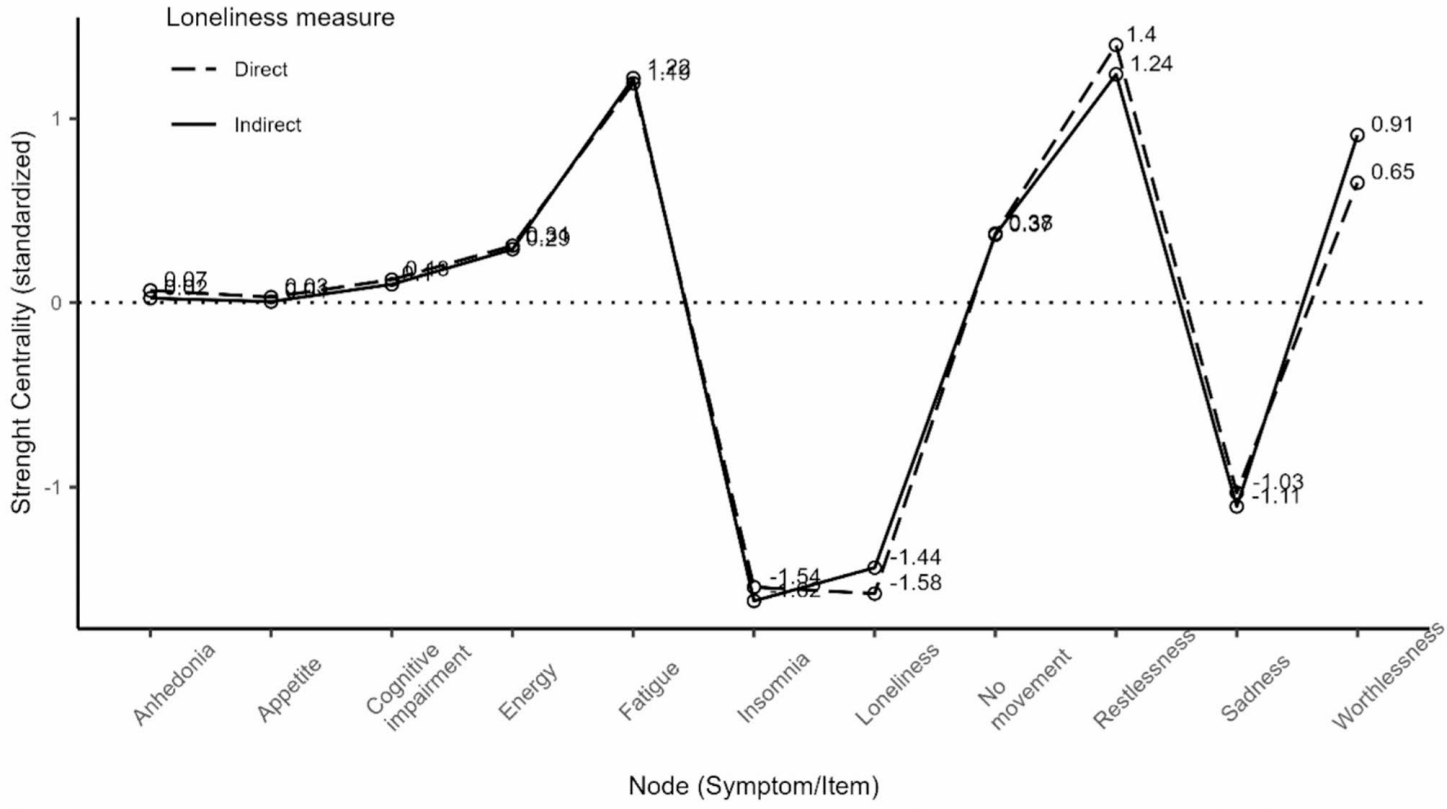
Standardized strength centrality scores for all nodes in the network.

At the same time, the analyses show that both direct and indirect loneliness has low centrality (Fig. 5) with negative standardized strength centrality coefficients (z scores: -1.58 for direct, -1.44 for indirect). This indicates that loneliness has less influence on other network elements than average. The strength centrality of loneliness ranks last (eleventh) in a network with indirect loneliness and ten (penultimate) in a network with direct loneliness. For bootstrapped strength difference tests see Figure S3.

The difference between standardized strength centrality of loneliness in the two networks was only 0.14 and statistically (as demonstrated by permutation tests of the difference) non-significant (*p* = 0.757), indicating similar low centrality in both networks^4^.

## Discussion

To our knowledge, this is the first empirical study investigating the links between loneliness and depressive symptoms among institutionally isolated adolescents, a group that is characterized by higher levels of loneliness and depression than adolescents in the general population.

The results indicate that loneliness is mainly related to affective depression symptoms such as sadness, worthlessness, anhedonia and restlessness. The sole somatic symptom weakly related to loneliness is a lack of energy, which occurs only when loneliness is measured directly. Thus, the relationships between loneliness and somatic symptoms are mediated through affective symptoms.

The connection between loneliness and affective symptoms is unsurprising, given that loneliness is commonly characterized as an affective reaction to the lack of a satisfactory interpersonal relationship. In this context, sadness (“the emotion associated with loss of contact with a positive reinforcer; that is, loss of a valued relationship, object or state”^123^) is most often viewed as one of the primary emotions experienced by lonely people^24,124–131^ and often used in defining loneliness^132,133^.

However, our study suggests that for institutionally isolated adolescents, emotions tied to loneliness go beyond sadness, encompassing worthlessness, anhedonia and restlessness as well. It is interesting that there were no substantial (statistically significant) differences in the relationship between loneliness and specific affective symptoms. This implies not only that in institutionally isolated adolescents loneliness is linked to a wide range of affective experiences, but also that no single emotion prominently dominates the others.

The link between loneliness and various negative emotions aligns with research showing that maladaptive emotion regulation may contribute to increased loneliness^22,101,134–140^. This insight also carries practical implications, helping us comprehend why focusing on biased emotional regulation offers a more promising approach to combating loneliness and yields better results than interventions of a more social nature, for example, targeting social support, social interaction and social skills^141^. Additionally, it clarifies why cognitive behavioral therapy CBT for depression, which targets cognitive biases, emotion regulation and behaviors perpetuating affective distress, is linked to decreased loneliness^142^. Consideration of emotions and their regulation should increase the effectiveness of interventions aimed at improving the psychosocial well-being of institutionally isolated young people.

One of the objectives of our study was also to examine the centrality of loneliness in the network of depressive symptoms. Contrary to previous findings (see Introduction section), our analysis shows that loneliness (both direct and indirect) has the lowest centrality within the network. This inconsistency may be due to the specifics of adolescents in institutional isolation. Note that none of the previous research analyzed data from such a group of respondents.

However, this contradiction may be methodological in nature. Previous research^143–145^ indicates that item relationships in a survey depend on their position, with items on the same or adjacent screens being more related than those on separate screens. However, higher inter-item correlations do not necessarily signify better data quality, as they may result from biases like nondifferentiated responding or other response biases^146^. These effects are based on the cognitive heuristic “near means related,” where participants perceive physically close items as related, even forming connections based on their spatial position^145^. Other visual aspects, such as the number of response options or grid usage, can also influence these effects^147^. Future empirical studies have yet to verify whether the different relation of lonelinness and depression in different studies is driven by a ‘nearly means related’ heuristic. To the authors’ knowledge, no study has compared these correlations based on scale positions within surveys.

An important aspect of our study was to determine how different measurement methods of loneliness (direct and indirect) affect the relationships between loneliness and depressive symptoms. Overall, our findings suggest that the method of measuring loneliness did not significantly impact these connections. Despite potential underestimation of loneliness^73^ due to social stigma^70–72^ and the possible influence of cognitive skills on estimating one’s own loneliness^74^our research indicates that direct measures of loneliness are as useful as indirect ones in the context of network analysis. This is useful information since the use of single items has practical advantages. It is generally associated with lower levels of mental fatigue which leads to higher response rates, a greater number of completed questionnaires and therefore leads to greater efficiency of a survey^148^. Shorter surveys may be especially beneficial for juvenile offenders, often characterized by deficits in cognitive capacity^149,150^.

### Practical implications

These findings have several practical implications, particularly for psychological and educational interventions targeting institutionally isolated adolescents. First, the strong links between loneliness and affective depressive symptoms (e.g., worthlessness, sadness, anhedonia, restlessness) highlight the importance of addressing maladaptive emotional patterns rather than focusing solely on enhancing social interactions. This supports the use of interventions that improve emotional regulation ‒ such as cognitive-behavioral therapy (CBT) ‒ as effective strategies for reducing loneliness in this population^141^. Emotion-focused treatments may be more beneficial than those aiming to directly increase social contact, which may not be feasible or effective in constrained institutional environments.

Other intervention strategies may also be relevant. Programs designed to enhance adolescents’ social and emotional competence, such as social and emotional learning (SEL)^151^have demonstrated comparable effectiveness to CBT in mitigating loneliness in youth populations^152^. These interventions may be particularly beneficial in structured settings like youth correctional centres, where opportunities for organic social interaction are limited.

In addition, group-based educational approaches ‒ such as Cooperative Learning (CL)^153^grounded in social interdependence theory ‒ have shown promise in improving peer relations, fostering positive emotional experiences, and reducing victimization^154–156^. CL encourages small-group collaboration around shared goals, which may help rebuild social trust and affiliation even in restrictive settings. However, careful management of group dynamics is essential, as some configurations may increase competition or reinforce social hierarchies.

Finally, given the high levels of emotion dysregulation and loneliness observed in this group, screening and preventive strategies should be prioritized alongside therapeutic interventions. Brief, low-burden assessments using direct measures of loneliness ‒ shown here to be psychometrically valid ‒ could be incorporated into institutional routines to help identify at-risk youth early. Taken together, these approaches highlight the need for integrated, emotion-focused prevention and intervention frameworks tailored to the unique psychological and social constraints of institutionally isolated adolescents.

### Limitations and future research directions

Some limitations should be considered. First, our cross-sectional data do not allow for determining the causal relationship between loneliness and depression symptoms. Future research should use longitudinal studies and temporal networks^157^ to better understand the dynamics between them, which could enhance therapeutic interventions.

Second, our study’s RCADS-MDD scale lacks suicide-related items, yet evidence links loneliness and adolescent suicide risk^51,59^. Since suicide is a significant of adolescent deaths^158,159^particularly for institutionally isolated individuals^160^future analyses should use depression questionnaires with suicide items, like the Patient Health Questionnaire Modified for Adolescents (PHQ-A).

Moreover, although the RCADS-MDD subscale captures most core depressive symptoms, it does not fully encompass the entire diagnostic criteria for major depressive disorder as defined by DSM-5. For example, symptoms such as excessive guilt and more detailed aspects of psychomotor changes were not comprehensively assessed. This limitation may have reduced the complexity and completeness of the estimated networks, potentially omitting relevant symptom-to-symptom associations. Future research should incorporate broader assessments to better reflect the full spectrum of depression severity.

Another important limitation of our study lies in the exclusive use of psychometric self-report measures, which are inherently vulnerable to response biases such as social desirability or inaccuracies in self-perception^161^. Although validated scales provide valuable insights, they may not fully capture the complexity of psychological phenomena like depression and loneliness. Recent research increasingly highlights biological underpinnings of both depression and loneliness, including elevated levels of inflammatory markers such as interleukin-6 (IL-6)^162–164^.

For instance, elevated IL-6 levels have been associated with both depressive symptoms and chronic loneliness, suggesting a potential psychoneuroimmunological pathway. Similarly, neuroimaging studies using functional MRI (fMRI) have identified altered brain activity patterns in lonely individuals and those with depression, particularly in regions related to emotion regulation and social cognition^165,166^. Future studies would benefit from integrating biological markers and neuroimaging techniques alongside psychometric assessments, which could offer a more comprehensive and multidimensional understanding of the mechanisms linking loneliness and depression.

Despite using validated and widely accepted psychometric instruments, it should be acknowledged that self-reported data are inherently subject to response biases, such as social desirability or inaccurate self-assessment^161^. To mitigate these limitations, future research could benefit from combining subjective reports with objective behavioral or physiological indicators, thereby enhancing the reliability and validity of findings. To enhance the reliability and validity of psychometric assessments, future clinical practice and research should adopt multimodal approaches. This could involve integrating subjective self-report measures with behavioral observations, physiological indicators (e.g., heart rate variability, cortisol levels), and biological markers (e.g., IL-6 concentrations). Such strategies could mitigate the impact of response biases and strengthen the replicability of findings.

Moreover, methodological concerns raised earlier regarding the potential influence of item proximity and scale presentation order warrant further empirical investigation. To the best of our knowledge, no previous studies have systematically investigated whether the positioning of loneliness and depression items within a survey influences the observed associations between them. Similarly, it remains unclear whether the order of scale presentation might affect the structure of loneliness–depression networks. In our study, all scales were presented in a fixed, non-randomized order, which may have introduced order effects. Although some studies have reported minimal or no order effects on depression-related measures^167,168^other research has found that presentation order can significantly influence results^169^.

Therefore, future research should aim to experimentally assess whether spatial proximity and scale presentation order affect the structure of loneliness–depression symptom networks. Such studies should utilize randomized or counterbalanced designs to minimize potential method biases. Furthermore, empirical work is needed to determine the magnitude and direction of these effects, particularly in child and adolescent populations, for which current evidence remains scarce. Clarifying these methodological influences would significantly enhance the validity and interpretability of network analyses examining loneliness and depression.

Another limitation concerns the lack of between-group network comparisons (e.g., by gender) and the exclusion of demographic variables from network estimation. Although demographic factors, such as gender or age, may influence the organization and strength of symptom networks, our sample size ‒ and particularly the small number of female participants ‒ did not allow for reliable subgroup analyses. Additionally, including background variables directly into the network estimation would have shifted the focus from symptom-to-symptom associations to predictor-outcome modeling, which was beyond the scope of the current study. Future research with larger and more balanced samples should investigate the potential moderating role of demographic and contextual variables in loneliness–depression symptom networks.

Finally, a key limitation of our study is the composition of the sample, which consisted exclusively of male adolescents placed in institutional isolation settings. Due to this sample structure and the low number of female participants, we were unable to conduct reliable between-group comparisons, for example by gender. Future research should include more diverse and balanced samples to examine potential moderation effects of demographic and contextual variables.

It is also important to note that the findings should not be generalized to the broader adolescent population, as our participants were not representative of community-based or clinical groups. Moreover, as already mentioned, the cross-sectional nature of the study limits causal interpretations. Many of the adolescents in our sample may have experienced prior adverse conditions ‒ such as trauma, emotional challenges, or socioeconomic difficulties ‒ that could contribute to elevated levels of loneliness independently of the institutional setting. Therefore, it is not possible to causally attribute the experience of loneliness solely to institutional isolation.

## Conclusions

In conclusion, this study demonstrates that loneliness in institutionally isolated adolescents is primarily related to affective depressive symptoms such as sadness, worthlessness, anhedonia, and restlessness. This connection is expected, as loneliness is typically considered an affective response to unsatisfactory interpersonal relationships. Importantly, our findings show that a wide range of emotions is associated with loneliness, with no single affective symptom emerging as dominant.

The observed associations between loneliness and negative emotional states align with existing evidence that maladaptive emotion regulation contributes to feelings of loneliness. This underscores the need for interventions focused on emotional processes, rather than solely enhancing social interaction. Interestingly, contrary to previous findings in general youth populations, loneliness emerged as the least central node in the depressive symptom network, which may reflect the unique characteristics of institutionally isolated adolescents or methodological aspects.

This is, to our knowledge, the first study to explore these dynamics in this population using both direct and indirect measures of loneliness and a network analytic approach. Our results further indicate that the choice of measurement strategy (direct vs. indirect) does not substantially affect the pattern of associations with depressive symptoms, supporting the use of brief, direct measures in time- and resource-limited institutional settings.

Beyond advancing theoretical understanding, the study offers practical insights for clinical and educational practice. By identifying affective symptoms as key intermediaries in the loneliness–depression link, it supports the development of emotion-focused preventive and therapeutic strategies tailored to the specific psychosocial constraints of institutional environments. Moreover, the findings suggest that short loneliness screeners may be useful for early identification of at-risk adolescents, offering potential applications in screening and prevention.

### Notes

1. We chose Spearman partial correlations rather than polychoric because the available research indicates that Spearman correlations produce more stable networks than polychoric correlations, especially when the sample size is small, items have few response options and are considerably skewed^116,121,170^.
2. Edge stability analysis exceeding the threshold of 0.5 (CS [cor = 0.7] = 0.52) indicated stable edge estimates.
3. Generally, the omnibus structure invariance test found no significant edge differences between the two networks (*M* = 0.07, *p* = 1), indicating marginal differences in correlation coefficient magnitudes (paths). The omnibus network comparison test revealed no significant global strength differences (*S* = 0.02, *p* = 0.834), suggesting equal connectivity levels between networks with direct and indirect loneliness. Thus, the paths in both networks are not significantly different.
4. Note that correlation stability coefficients for strength centrality exceeded the recommended 0.5 cut-off (direct: CS [cor = 0.7] = 0.52; indirect: CS [cor = 0.7] = 0.59), suggesting stable and reliable symptom classification for the strength centrality index for both networks.

## Electronic supplementary material

Below is the link to the electronic supplementary material.

Supplementary Material 1

## Data Availability

The research data and statistical analysis codes supporting the findings of this manuscript are available from the corresponding author upon reasonable request.
