# Supplementary material for: Loneliness and depressive symptoms in institutionally isolated Polish adolescents explored through network psychometrics
Source: Sci Rep. 2025 Jul 1;15:21444. doi: 10.1038/s41598-025-06541-5 (PMC12214851; doi:10.1038/s41598-025-06541-5)
Supplement: Supplementary file 1 — Supplementary Material 1 [file 41598_2025_6541_MOESM1_ESM.docx]

**Supplementary Materials**

Table S1. Edge weights from regularized Gaussian Graphical Models (GGM): direct loneliness (above diagonal) and indirect loneliness (DJGLS; below diagonal)

|  | 1 | 2 | 3 | 4 | 5 | 6 | 7 | 8 | 9 | 10 | 11 |
| --- | --- | --- | --- | --- | --- | --- | --- | --- | --- | --- | --- |
| 1. Sadness | 0.00 | 0.23 | 0.01 | 0.03 | 0.01 | 0.11 | 0 | 0 | 0 | 0.12 | 0.19 |
| 1. Anhedonia | 0.23 | 0 | 0.05 | 0 | 0.08 | 0 | 0.01 | 0.25 | 0.1 | 0.05 | 0.11 |
| 1. Insomnia | 0.01 | 0.05 | 0 | 0.24 | 0.14 | 0.08 | 0.01 | 0 | 0.06 | 0.03 | 0 |
| 1. Appetite | 0.03 | 0 | 0.24 | 0 | 0.03 | 0.11 | 0.20 | 0.08 | 0.04 | 0.15 | 0 |
| 1. Energy | 0.01 | 0.08 | 0.14 | 0.03 | 0 | 0.27 | 0 | 0.05 | 0.17 | 0.16 | <0.01 |
| 1. Fatigue | 0.11 | 0 | 0.08 | 0.11 | 0.27 | 0 | 0.25 | 0 | 0.16 | 0.09 | 0 |
| 1. Cognitive impairment | 0.00 | 0.01 | 0.01 | 0.20 | 0 | 0.25 | 0 | 0.18 | 0.19 | 0.07 | 0 |
| 1. Worthlessness | 0.00 | 0.24 | 0 | 0.08 | 0.05 | 0 | 0.17 | 0 | 0.09 | 0.17 | 0.15 |
| 1. No movement | 0.00 | 0.1 | 0.06 | 0.04 | 0.17 | 0.16 | 0.19 | 0.09 | 0 | 0.11 | 0 |
| 1. Restlessness | 0.12 | 0.05 | 0.03 | 0.15 | 0.16 | 0.09 | 0.07 | 0.16 | 0.11 | 0 | 0.15 |
| 1. Loneliness | 0.20 | 0.12 | 0 | 0 | 0 | 0 | 0 | 0.22 | 0 | 0.11 | 0 |

*Note*. Edge weights represent regularized partial correlations estimated using Gaussian graphical models (GGM) with LASSO regularization and EBIC model selection. Regularization shrinks trivial associations to zero, retaining only the most robust connections (see Statistical Analyses – Network estimation for details). Classical significance testing was not applied; only edges surviving regularization are presented.

Figure S1. Plot of the bootstrapped difference tests (α = .05) for edge-weights.


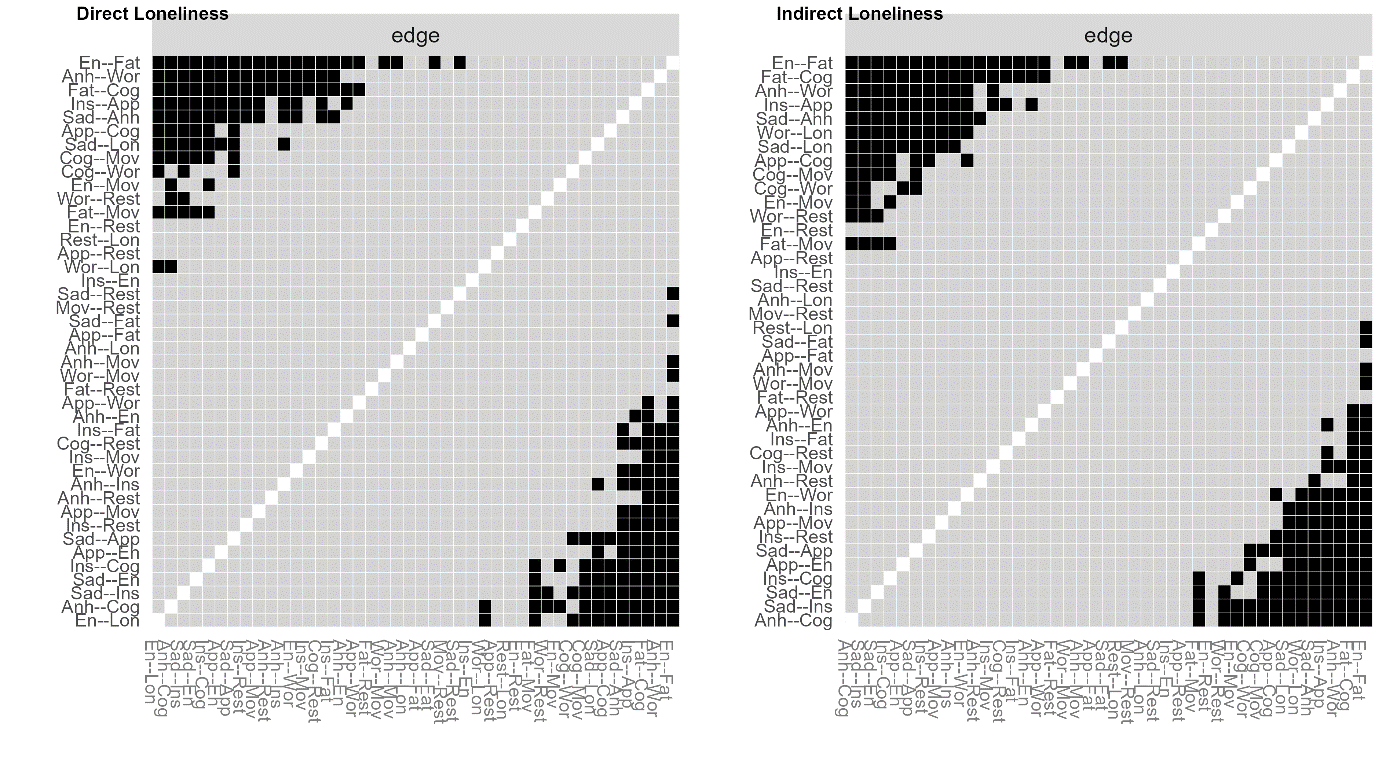


*Note*. Gray boxes indicate that there is no difference between edge-weights, whereas black boxes indicate significant difference (α = 0.05)

Figure S2. Delta network of edge weights that differ between networks with direct and indirect loneliness.


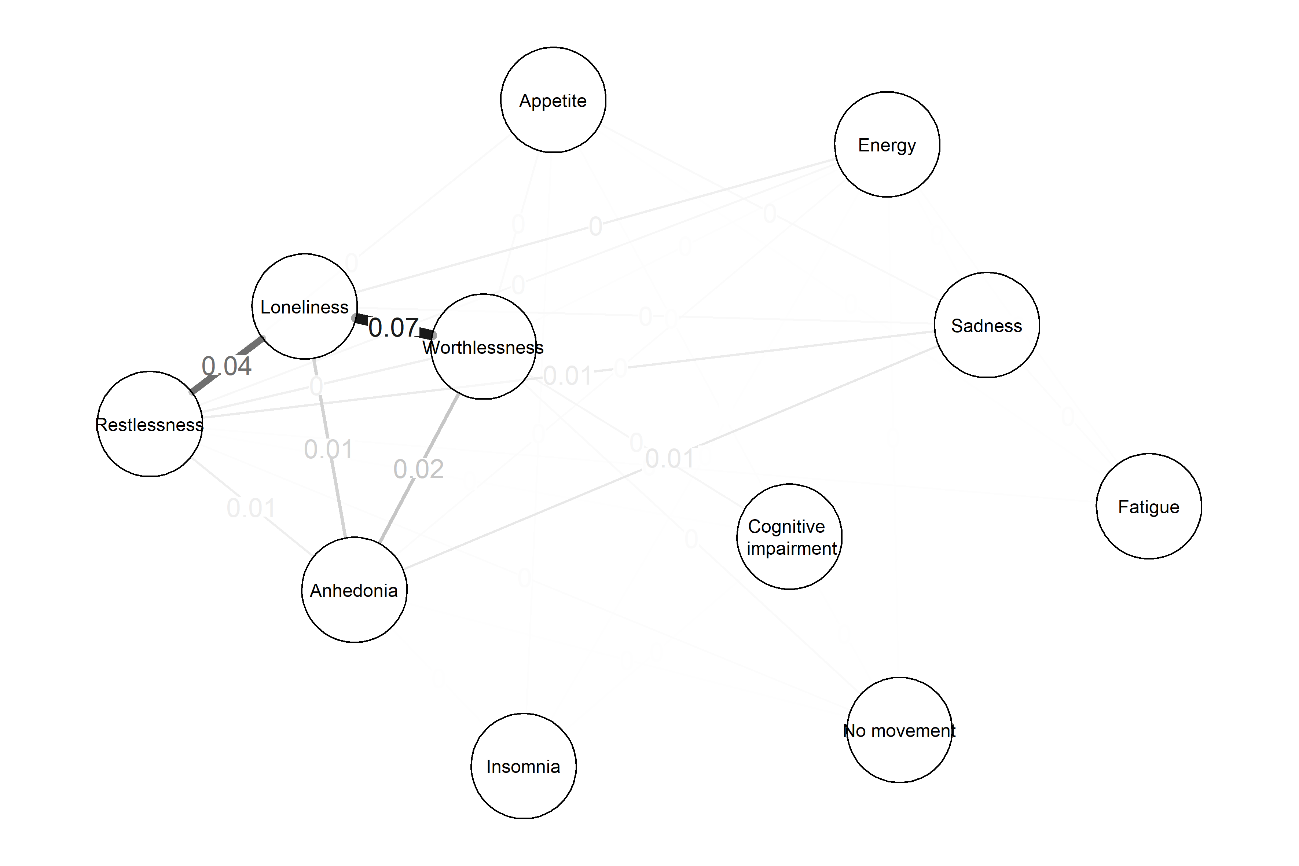


Note. Thicker edges represent a greater difference between a particular edge weight in two networks. Values indicate the difference between the edges. None of the differences are statistically significant (at *p* = 0.05).

Figure S3. Nonparametric bootstraped difference test (α = .05) for strength.


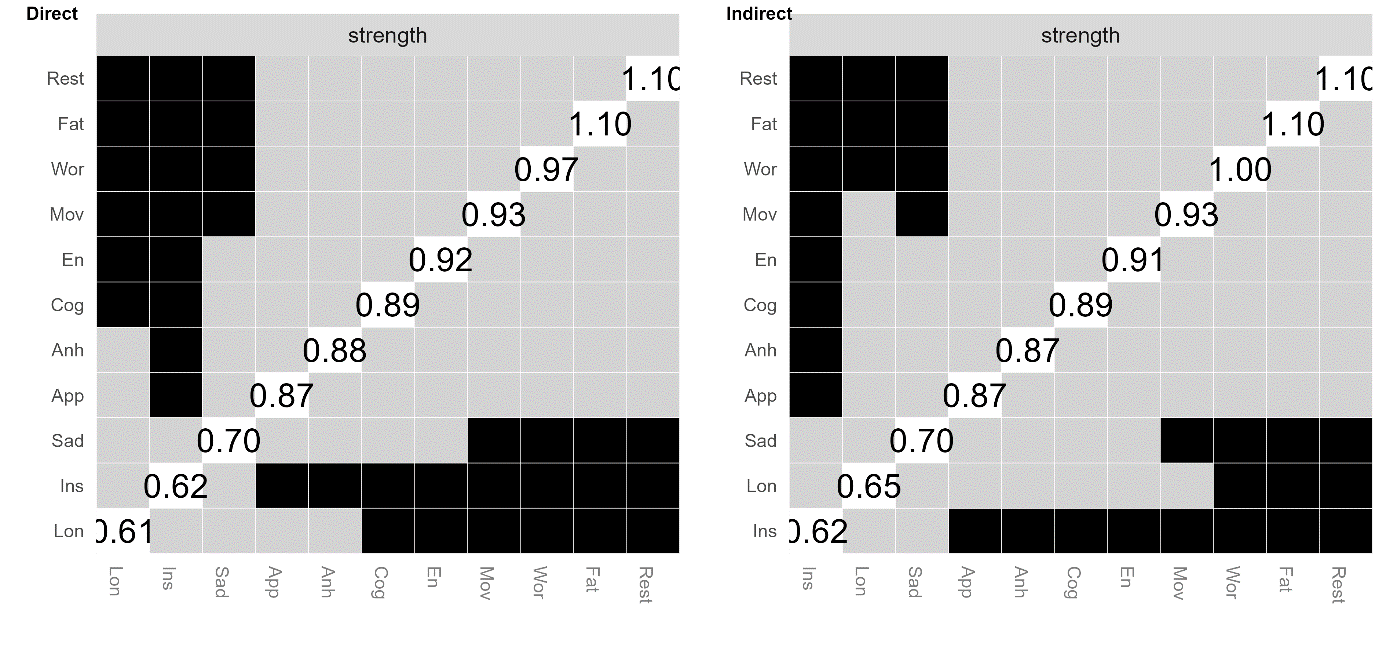


*Note*. Gray boxes indicate that there is no difference between nodes, whereas black boxes indicate significant difference (α = 0.05). The values reported on the diagonal represent the (unstandarized) strength values of each node.
